# 2-(4-Methyl­phen­yl)-1-(phenyl­sulfon­yl)propan-2-ol

**DOI:** 10.1107/S1600536811039894

**Published:** 2011-10-05

**Authors:** Bao-Jun Shi, Liang-Zhu Huang, You-Qiang Li, Chang-Mei Si, Zhen-Ting Du

**Affiliations:** a College of Plant Protection, Northwest A&F University, Yangling 712100, People’s Republic of China; b College of Science, Northwest A&F University, Yangling 712100, People’s Republic of China

## Abstract

The title compound, C_16_H_18_O_3_S, features a U-shape mol­ecular structure with a dihedral angle between the terminal benzene rings of 20.8 (1)°. An intra­molecular O—H⋯O hydrogen bond helps to stabilize the mol­ecular structure. Inter­molecular classical O—H⋯O and weak C—H⋯O hydrogen bonding is present in the crystal structure.

## Related literature

For the use of organic sulfones as inter­mediates in organic synthesis, see: Consiglio *et al.* (1983 ▶); Wenkert *et al.* (1983 ▶); Trost (1991 ▶). For related structures, see: Gu *et al.* (2004 ▶); Garst *et al.* (2006 ▶); Ding *et al.*, (2009 ▶); Groszek *et al.* (2006 ▶); Shi *et al.* (2011 ▶). For background to our program to synthesis new herbicide derivatives, see: Du *et al.* (2011 ▶).
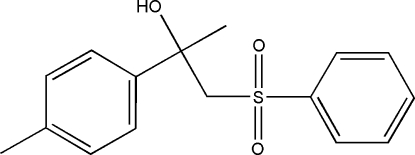


## Experimental

### 

#### Crystal data

C_16_H_18_O_3_S
*M* *_r_* = 290.36
Orthorhombic, 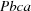

*a* = 15.6696 (14) Å
*b* = 11.7501 (11) Å
*c* = 15.9042 (16) Å
*V* = 2928.3 (5) Å^3^
*Z* = 8
Mo *K*α radiation
μ = 0.23 mm^−1^
*T* = 298 K
0.38 × 0.29 × 0.21 mm

#### Data collection

Bruker SMART 1000 CCD area-detector diffractometer
Absorption correction: multi-scan (*SADABS*; Sheldrick, 1996 ▶) *T*_min_ = 0.919, *T*_max_ = 0.954
13679 measured reflections
2578 independent reflections
1590 reflections with *I* > 2σ(*I*)
*R*_int_ = 0.055

#### Refinement

*R*[*F* ^2^ > 2σ(*F* ^2^)] = 0.044
*wR*(*F* ^2^) = 0.141
*S* = 1.08
2578 reflections
183 parameters
H-atom parameters constrained
Δρ_max_ = 0.27 e Å^−3^
Δρ_min_ = −0.30 e Å^−3^

### 

Data collection: *SMART* (Bruker, 2007 ▶); cell refinement: *SAINT* (Bruker, 2007 ▶); data reduction: *SAINT*; program(s) used to solve structure: *SHELXTL* (Sheldrick, 2008 ▶); program(s) used to refine structure: *SHELXTL*; molecular graphics: *SHELXTL*; software used to prepare material for publication: *SHELXTL*.

## Supplementary Material

Crystal structure: contains datablock(s) I, global. DOI: 10.1107/S1600536811039894/xu5336sup1.cif

Structure factors: contains datablock(s) I. DOI: 10.1107/S1600536811039894/xu5336Isup2.hkl

Supplementary material file. DOI: 10.1107/S1600536811039894/xu5336Isup3.cml

Additional supplementary materials:  crystallographic information; 3D view; checkCIF report

## Figures and Tables

**Table 1:** Hydrogen-bond geometry (Å, °)

*D*—H⋯*A*	*D*—H	H⋯*A*	*D*⋯*A*	*D*—H⋯*A*
O1—H1⋯O3	0.82	2.14	2.848 (3)	144
O1—H1⋯O3^i^	0.82	2.45	3.103 (3)	137
C15—H15⋯O2^ii^	0.93	2.48	3.403 (5)	173

Symmetry codes: (i) 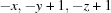; (ii) 
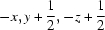
.

**Fig. 1.**
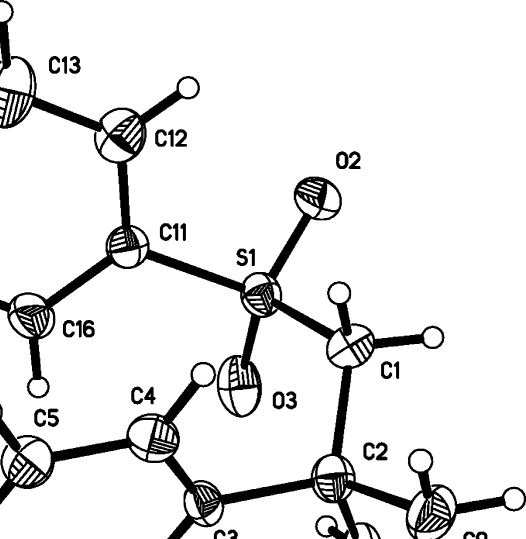
The molecular structure of the title compound with the atom numbering scheme. Displacement ellipsoids are drawn at the 30% probability level.

**Fig. 2.**
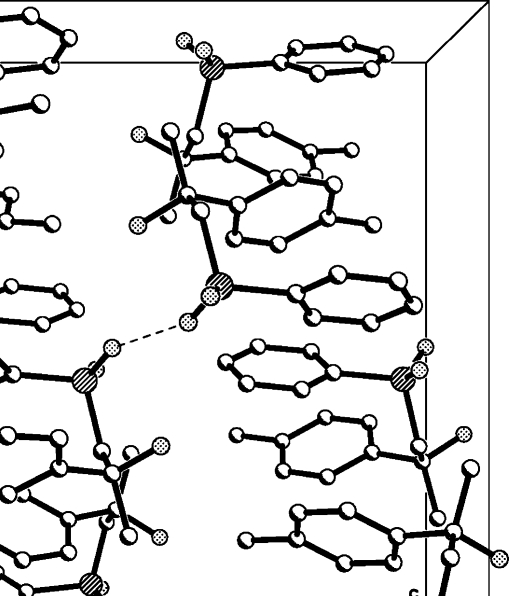
The molecular packing of (I) viewed along the a axis, with hydrogen bonds shown as dashed lines.

## supplementary crystallographic information

### Comment

Organic sulfones have been proven as good intermediates in organic synthesis (Consiglio *et al.*, 1983; Wenkert *et al.*, 1983; Trost 1991). As a result of our program of synthesis of new herbcide derivatives (Du *et al.*, 2011), we obtained a intermediate compound C_16_H_18_O_3_S (I) in the synthesis and structure are reported here. There are two benzene rings in the title compound and they exhibit face-to-face conformation. The dihedral angle between the two benzene rings is 20.8 (1)°. The molecules of I are crystalized in *Pbca* space group which differs from that of 2-methoxy-4-methyl-1-(1-(phenylsulfonyl)propan-2-yl)benzene (*P*2~1~/*c*, Shi *et al.*, 2011). In the crystal structure there is an intramolecular C—H···O hydrogen-bonding interaction (Table 1) which is benefical to the stabilization of the packing, and whose symmetry code is defined as -*x*, -*y* + 1, -*z* + 1.

### Experimental

A mixture of 2-methyl-2-(*p*-tolyl)oxirane (148 mg, 1 mmol) and sodium benzenesulfinate (246 mg, 1.5 mmol) in dry DMF (3 ml) was stirred over night at 80°C. When the reaction was completed, 2 ml water was added to the reaction mixture to quench reaction, then was extracted with ethyl acetate (20 ml × 3). The ethyl acetate layers were combined and washed by 20 ml water, then 15 ml saturated sodium chloride and dried over anhydrous sodium sulfate. The solution was evapourated and the residue was separated on silica gel column chromatography with a gradient of petroleum ether and ethyl acetate as eluent to yield 348 mg the title compound. The compound was then dissolved in methanol, and colorless crystals were formed on slow evaporation at room temperature over one week.

### Refinement

H atoms were placed in idealized positions (C—H = 0.93–0.97 Å, O—H = 0.82 Å) and refined as riding atoms with U_iso_(H) = 1.5U_eq_(O,C) for hydroxyl and methyl H atoms and 1.2U_eq_(C) for the others.

### Crystal data

**Table:**

C_16_H_18_O_3_S	*D*_x_ = 1.317 Mg m^−^^3^
*M**_r_* = 290.36	Melting point: 392 K
Orthorhombic, *P**b**c**a*	Mo *K*α radiation, λ = 0.71073 Å
Hall symbol: -P 2ac 2ab	Cell parameters from 2803 reflections
*a* = 15.6696 (14) Å	θ = 2.5–23.6°
*b* = 11.7501 (11) Å	µ = 0.23 mm^−^^1^
*c* = 15.9042 (16) Å	*T* = 298 K
*V* = 2928.3 (5) Å^3^	Block, colorless
*Z* = 8	0.38 × 0.29 × 0.21 mm
*F*(000) = 1232	

### Data collection

**Table:**

Bruker SMART 1000 CCD area-detector diffractometer	2578 independent reflections
Radiation source: fine-focus sealed tube	1590 reflections with *I* > 2σ(*I*)
graphite	*R*_int_ = 0.055
φ and ω scans	θ_max_ = 25.0°, θ_min_ = 2.5°
Absorption correction: multi-scan (*SADABS*; Sheldrick, 1996)	*h* = −18→18
*T*_min_ = 0.919, *T*_max_ = 0.954	*k* = −7→13
13679 measured reflections	*l* = −18→18

### Refinement

**Table:**

Refinement on *F*^2^	Primary atom site location: structure-invariant direct methods
Least-squares matrix: full	Secondary atom site location: difference Fourier map
*R*[*F*^2^ > 2σ(*F*^2^)] = 0.044	Hydrogen site location: inferred from neighbouring sites
*wR*(*F*^2^) = 0.141	H-atom parameters constrained
*S* = 1.08	*w* = 1/[σ^2^(*F*_o_^2^) + (0.054*P*)^2^ + 2.2452*P*] where *P* = (*F*_o_^2^ + 2*F*_c_^2^)/3
2578 reflections	(Δ/σ)_max_ < 0.001
183 parameters	Δρ_max_ = 0.27 e Å^−^^3^
0 restraints	Δρ_min_ = −0.30 e Å^−^^3^

### Special details

**Table:**

Geometry. All e.s.d.'s (except the e.s.d. in the dihedral angle between two l.s. planes) are estimated using the full covariance matrix. The cell e.s.d.'s are taken into account individually in the estimation of e.s.d.'s in distances, angles and torsion angles; correlations between e.s.d.'s in cell parameters are only used when they are defined by crystal symmetry. An approximate (isotropic) treatment of cell e.s.d.'s is used for estimating e.s.d.'s involving l.s. planes.
Refinement. Refinement of *F*^2^ against ALL reflections. The weighted *R*-factor *wR* and goodness of fit *S* are based on *F*^2^, conventional *R*-factors *R* are based on *F*, with *F* set to zero for negative *F*^2^. The threshold expression of *F*^2^ > σ(*F*^2^) is used only for calculating *R*-factors(gt) *etc*. and is not relevant to the choice of reflections for refinement. *R*-factors based on *F*^2^ are statistically about twice as large as those based on *F*, and *R*- factors based on ALL data will be even larger.

### Fractional atomic coordinates and isotropic or equivalent isotropic displacement parameters (Å^2^)

**Table:**

	*x*	*y*	*z*	*U*_iso_*/*U*_eq_	
O1	0.16491 (15)	0.5125 (2)	0.51682 (13)	0.0541 (7)	
H1	0.1135	0.5015	0.5118	0.081*	
O2	0.05450 (17)	0.2052 (2)	0.41610 (15)	0.0608 (7)	
O3	0.01924 (14)	0.4061 (2)	0.44403 (15)	0.0607 (7)	
S1	0.07045 (5)	0.32361 (7)	0.40083 (5)	0.0424 (3)	
C1	0.17920 (19)	0.3470 (3)	0.4275 (2)	0.0441 (8)	
H1A	0.1918	0.3022	0.4772	0.053*	
H1B	0.2140	0.3171	0.3822	0.053*	
C2	0.2081 (2)	0.4699 (3)	0.44465 (19)	0.0428 (8)	
C3	0.19648 (19)	0.5463 (3)	0.36837 (19)	0.0376 (7)	
C4	0.2366 (2)	0.5198 (3)	0.2928 (2)	0.0446 (8)	
H4	0.2691	0.4537	0.2892	0.053*	
C5	0.2294 (2)	0.5888 (3)	0.2233 (2)	0.0473 (9)	
H5	0.2564	0.5680	0.1735	0.057*	
C6	0.1827 (2)	0.6890 (3)	0.2262 (2)	0.0453 (8)	
C7	0.1425 (2)	0.7151 (3)	0.3009 (2)	0.0477 (9)	
H7	0.1101	0.7812	0.3043	0.057*	
C8	0.1492 (2)	0.6455 (3)	0.3710 (2)	0.0448 (8)	
H8	0.1215	0.6659	0.4205	0.054*	
C9	0.3024 (2)	0.4680 (3)	0.4693 (2)	0.0586 (10)	
H9A	0.3211	0.5440	0.4815	0.088*	
H9B	0.3354	0.4375	0.4238	0.088*	
H9C	0.3097	0.4212	0.5183	0.088*	
C10	0.1751 (3)	0.7661 (3)	0.1507 (2)	0.0644 (11)	
H10A	0.1555	0.8397	0.1684	0.097*	
H10B	0.1352	0.7341	0.1115	0.097*	
H10C	0.2299	0.7735	0.1243	0.097*	
C11	0.06017 (19)	0.3471 (3)	0.2917 (2)	0.0402 (8)	
C12	0.0856 (2)	0.2625 (3)	0.2375 (2)	0.0557 (10)	
H12	0.1085	0.1950	0.2581	0.067*	
C13	0.0767 (3)	0.2792 (4)	0.1524 (3)	0.0721 (13)	
H13	0.0935	0.2224	0.1151	0.086*	
C14	0.0435 (3)	0.3785 (5)	0.1222 (3)	0.0742 (13)	
H14	0.0378	0.3889	0.0645	0.089*	
C15	0.0186 (3)	0.4630 (4)	0.1765 (3)	0.0698 (12)	
H15	−0.0036	0.5307	0.1555	0.084*	
C16	0.0264 (2)	0.4476 (3)	0.2624 (2)	0.0546 (9)	
H16	0.0092	0.5042	0.2996	0.065*	

### Atomic displacement parameters (Å^2^)

**Table:**

	*U*^11^	*U*^22^	*U*^33^	*U*^12^	*U*^13^	*U*^23^
O1	0.0580 (15)	0.0663 (17)	0.0380 (13)	−0.0058 (13)	0.0003 (11)	−0.0134 (12)
O2	0.0805 (18)	0.0463 (15)	0.0555 (15)	−0.0190 (13)	−0.0042 (13)	0.0066 (12)
O3	0.0468 (14)	0.0772 (18)	0.0582 (15)	0.0029 (13)	0.0069 (12)	−0.0265 (14)
S1	0.0442 (5)	0.0438 (5)	0.0392 (5)	−0.0035 (4)	0.0028 (4)	−0.0042 (4)
C1	0.0426 (19)	0.049 (2)	0.0409 (18)	0.0050 (16)	0.0008 (15)	0.0056 (16)
C2	0.0438 (19)	0.046 (2)	0.0384 (18)	−0.0014 (16)	0.0013 (14)	−0.0037 (16)
C3	0.0355 (17)	0.0355 (18)	0.0417 (18)	−0.0049 (14)	−0.0042 (14)	−0.0056 (15)
C4	0.0432 (19)	0.042 (2)	0.0488 (19)	0.0049 (16)	0.0036 (15)	−0.0011 (17)
C5	0.048 (2)	0.049 (2)	0.045 (2)	−0.0008 (17)	0.0041 (16)	0.0005 (17)
C6	0.0387 (18)	0.046 (2)	0.051 (2)	−0.0055 (16)	−0.0084 (16)	0.0015 (17)
C7	0.0421 (19)	0.039 (2)	0.062 (2)	0.0009 (16)	−0.0043 (17)	−0.0049 (18)
C8	0.0430 (18)	0.044 (2)	0.047 (2)	−0.0028 (17)	0.0003 (15)	−0.0084 (17)
C9	0.048 (2)	0.071 (3)	0.057 (2)	−0.0061 (19)	−0.0154 (17)	0.007 (2)
C10	0.067 (3)	0.066 (3)	0.060 (2)	0.002 (2)	−0.006 (2)	0.012 (2)
C11	0.0367 (17)	0.042 (2)	0.0421 (18)	−0.0008 (15)	−0.0010 (14)	0.0012 (15)
C12	0.057 (2)	0.063 (2)	0.047 (2)	0.0100 (19)	0.0081 (18)	−0.0064 (19)
C13	0.063 (3)	0.106 (4)	0.048 (2)	0.003 (3)	0.011 (2)	−0.016 (3)
C14	0.064 (3)	0.110 (4)	0.048 (2)	−0.021 (3)	−0.002 (2)	0.020 (3)
C15	0.065 (3)	0.068 (3)	0.077 (3)	−0.020 (2)	−0.025 (2)	0.030 (3)
C16	0.052 (2)	0.044 (2)	0.068 (3)	−0.0073 (18)	−0.0150 (19)	0.0010 (19)

### Geometric parameters (Å, °)

**Table:**

O1—C2	1.424 (4)	C7—H7	0.9300
O1—H1	0.8200	C8—H8	0.9300
O2—S1	1.435 (2)	C9—H9A	0.9600
O3—S1	1.433 (2)	C9—H9B	0.9600
S1—C11	1.764 (3)	C9—H9C	0.9600
S1—C1	1.778 (3)	C10—H10A	0.9600
C1—C2	1.538 (4)	C10—H10B	0.9600
C1—H1A	0.9700	C10—H10C	0.9600
C1—H1B	0.9700	C11—C12	1.375 (5)
C2—C3	1.520 (4)	C11—C16	1.376 (5)
C2—C9	1.528 (4)	C12—C13	1.375 (5)
C3—C8	1.382 (4)	C12—H12	0.9300
C3—C4	1.392 (4)	C13—C14	1.365 (6)
C4—C5	1.375 (4)	C13—H13	0.9300
C4—H4	0.9300	C14—C15	1.373 (6)
C5—C6	1.387 (5)	C14—H14	0.9300
C5—H5	0.9300	C15—C16	1.384 (5)
C6—C7	1.378 (5)	C15—H15	0.9300
C6—C10	1.509 (5)	C16—H16	0.9300
C7—C8	1.387 (5)		
			
C2—O1—H1	109.5	C3—C8—C7	120.9 (3)
O3—S1—O2	118.50 (16)	C3—C8—H8	119.5
O3—S1—C11	108.34 (15)	C7—C8—H8	119.5
O2—S1—C11	107.58 (15)	C2—C9—H9A	109.5
O3—S1—C1	108.52 (15)	C2—C9—H9B	109.5
O2—S1—C1	106.05 (15)	H9A—C9—H9B	109.5
C11—S1—C1	107.35 (15)	C2—C9—H9C	109.5
C2—C1—S1	118.0 (2)	H9A—C9—H9C	109.5
C2—C1—H1A	107.8	H9B—C9—H9C	109.5
S1—C1—H1A	107.8	C6—C10—H10A	109.5
C2—C1—H1B	107.8	C6—C10—H10B	109.5
S1—C1—H1B	107.8	H10A—C10—H10B	109.5
H1A—C1—H1B	107.1	C6—C10—H10C	109.5
O1—C2—C3	112.3 (3)	H10A—C10—H10C	109.5
O1—C2—C9	104.9 (3)	H10B—C10—H10C	109.5
C3—C2—C9	109.2 (3)	C12—C11—C16	121.3 (3)
O1—C2—C1	109.5 (3)	C12—C11—S1	118.5 (3)
C3—C2—C1	112.2 (3)	C16—C11—S1	120.2 (3)
C9—C2—C1	108.4 (3)	C11—C12—C13	119.0 (4)
C8—C3—C4	117.2 (3)	C11—C12—H12	120.5
C8—C3—C2	122.6 (3)	C13—C12—H12	120.5
C4—C3—C2	120.2 (3)	C14—C13—C12	120.5 (4)
C5—C4—C3	121.6 (3)	C14—C13—H13	119.8
C5—C4—H4	119.2	C12—C13—H13	119.8
C3—C4—H4	119.2	C13—C14—C15	120.3 (4)
C4—C5—C6	121.1 (3)	C13—C14—H14	119.8
C4—C5—H5	119.4	C15—C14—H14	119.8
C6—C5—H5	119.4	C14—C15—C16	120.1 (4)
C7—C6—C5	117.3 (3)	C14—C15—H15	119.9
C7—C6—C10	121.1 (3)	C16—C15—H15	119.9
C5—C6—C10	121.6 (3)	C11—C16—C15	118.7 (4)
C6—C7—C8	121.8 (3)	C11—C16—H16	120.6
C6—C7—H7	119.1	C15—C16—H16	120.6
C8—C7—H7	119.1		
			
O3—S1—C1—C2	−32.7 (3)	C10—C6—C7—C8	−179.6 (3)
O2—S1—C1—C2	−161.1 (2)	C4—C3—C8—C7	0.0 (5)
C11—S1—C1—C2	84.2 (3)	C2—C3—C8—C7	177.7 (3)
S1—C1—C2—O1	64.0 (3)	C6—C7—C8—C3	−0.3 (5)
S1—C1—C2—C3	−61.4 (3)	O3—S1—C11—C12	−163.9 (3)
S1—C1—C2—C9	177.9 (2)	O2—S1—C11—C12	−34.7 (3)
O1—C2—C3—C8	0.2 (4)	C1—S1—C11—C12	79.1 (3)
C9—C2—C3—C8	−115.7 (3)	O3—S1—C11—C16	15.4 (3)
C1—C2—C3—C8	124.0 (3)	O2—S1—C11—C16	144.6 (3)
O1—C2—C3—C4	177.9 (3)	C1—S1—C11—C16	−101.7 (3)
C9—C2—C3—C4	61.9 (4)	C16—C11—C12—C13	−0.2 (5)
C1—C2—C3—C4	−58.3 (4)	S1—C11—C12—C13	179.0 (3)
C8—C3—C4—C5	−0.3 (5)	C11—C12—C13—C14	0.3 (6)
C2—C3—C4—C5	−178.1 (3)	C12—C13—C14—C15	0.0 (6)
C3—C4—C5—C6	1.0 (5)	C13—C14—C15—C16	−0.5 (6)
C4—C5—C6—C7	−1.2 (5)	C12—C11—C16—C15	−0.2 (5)
C4—C5—C6—C10	179.3 (3)	S1—C11—C16—C15	−179.5 (3)
C5—C6—C7—C8	0.9 (5)	C14—C15—C16—C11	0.6 (6)

### Hydrogen-bond geometry (Å, °)

**Table:**

*D*—H···*A*	*D*—H	H···*A*	*D*···*A*	*D*—H···*A*
O1—H1···O3	0.82	2.14	2.848 (3)	144
O1—H1···O3^i^	0.82	2.45	3.103 (3)	137
C15—H15···O2^ii^	0.93	2.48	3.403 (5)	173

Symmetry codes: (i) −*x*, −*y*+1, −*z*+1; (ii) −*x*, *y*+1/2, −*z*+1/2.
